# Mild drought in the vegetative stage induces phenotypic, gene expression, and DNA methylation plasticity in Arabidopsis but no transgenerational effects

**DOI:** 10.1093/jxb/eraa132

**Published:** 2020-03-13

**Authors:** Tom J M Van Dooren, Amanda Bortolini Silveira, Elodie Gilbault, José M Jiménez-Gómez, Antoine Martin, Liên Bach, Sébastien Tisné, Leandro Quadrana, Olivier Loudet, Vincent Colot

**Affiliations:** 1 CNRS - UMR 7618 Institute of Ecology and Environmental Sciences (iEES) Paris, Sorbonne University, Case 237, 4, place Jussieu, 75005 Paris, France; 2 Institut de Biologie de l’Ecole Normale Supérieure, (IBENS), Ecole Normale Supérieure, Centre National de la Recherche Scientifique (CNRS), Institut National de la Santé et de la Recherche Médicale (INSERM), PSL Université Paris, Paris, France; 3 Institut Jean-Pierre Bourgin, INRAE, AgroParisTech, Université Paris-Saclay, 78000 Versailles, France; 4 Hong Kong Baptist University, China

**Keywords:** Arabidopsis, drought, epigenetics, maternal effects, methylation, plasticity, transgenerational effects

## Abstract

There is renewed interest in whether environmentally induced changes in phenotypes can be heritable. In plants, heritable trait variation can occur without DNA sequence mutations through epigenetic mechanisms involving DNA methylation. However, it remains unknown whether this alternative system of inheritance responds to environmental changes and if it can provide a rapid way for plants to generate adaptive heritable phenotypic variation. To assess potential transgenerational effects induced by the environment, we subjected four natural accessions of *Arabidopsis thaliana* together with the reference accession Col-0 to mild drought in a multi-generational experiment. As expected, plastic responses to drought were observed in each accession, as well as a number of intergenerational effects of the parental environments. However, after an intervening generation without stress, except for a very few trait-based parental effects, descendants of stressed and non-stressed plants were phenotypically indistinguishable irrespective of whether they were grown in control conditions or under water deficit. In addition, genome-wide analysis of DNA methylation and gene expression in Col-0 demonstrated that, while mild drought induced changes in the DNA methylome of exposed plants, these variants were not inherited. We conclude that mild drought stress does not induce transgenerational epigenetic effects.

## Teaser

A comprehensive phenotypic analysis in five Arabidopsis accessions combined with methylome and transcriptome analyses in the Col-0 reference accession indicate plasticity to mild drought but no transgenerational effects or inherited stress memory.

## Introduction

Being sessile organisms, plants are exposed to environmental conditions from which they cannot escape. Stress responses to these conditions are multivariate (Claeys and Inzé, 2013), involving, for example, changes in stomatal conductance or amounts of protective proteins. The presence of stress can be inferred in an integrative manner by comparing plant growth rates in different conditions (Claeys *et al.*, 2014), allowing the identification of the presence of a stress response.

Stressors can not only have a profound impact on the growth and development of exposed individuals but also on their offspring. These parental (typically maternal) environmental effects have been well documented (Blödner *et al.*, 2007; Galloway and Etterson, 2007; Donohue, 2009; Herman and Sultan, 2011; Crisp *et al.*, 2016; Van Dooren *et al.*, 2016). However, when developing offspring (including the germ cells that produce the embryo) experience the same stressors as their parents, a transfer of information between generations is not necessary for an effect in the offspring. This multigenerational exposure (Skinner, 2008) has prompted the distinction between intergenerational and transgenerational effects (Heard and Martienssen, 2014). Intergenerational effects refer to when the environment that provokes the effects is not only experienced by the parents but also by the germ cells or, in the case of female mammals for example, by the fetuses they carry. Transgenerational effects occur in descendants that have not experienced the environmental stressor during any phase of their development (Heard and Martienssen, 2014), which implies that information regarding the stressor is transmitted from previous generations. This distinction is not always respected or made explicit. Another way of characterizing parental effects is by referring to transgenerational plasticity (Mousseau and Dingle, 1991; Herman and Sultan, 2011). This terminology assumes parental effects in offspring to be responses to environmental cues experienced by the parents. Confusingly, such effects can be intergenerational. When so-called memory effects in stress responses (Lämke and Bäurle, 2017) span different generations, they can again involve intergenerational as well as transgenerational effects. Trait-based parental effects (Kirkpatrick and Lande, 1989) occur when phenotypes of parents determine phenotypes of offspring. These effects can be either intergenerational or transgenerational. They seem a natural framework within which to assess phenotypic stress responses, because they can be used to investigate the determination of traits such as individual growth rates or other stress responses across generations. Trait-based parental effects can decay rapidly or persist due to evolutionary momentum, even without continual environmental change (Kirkpatrick and Lande, 1989).

Few experimental studies have been conducted to determine if a phenotypic memory of environmental stress can persist over multiple generations, and whether such effects are truly transgenerational. In a study by Whittle *et al.* (2009), genetically identical Arabidopsis lines grown under mild heat during the reproductive phase (from bolting onward) over two generations and then grown under normal conditions for an additional generation produced progeny with an ameliorated response to heat compared to control progeny from non-treated lines. However, as heat was applied during reproductive growth, the gametes and the developing seeds were also exposed to the environmental stressor, and therefore, intergenerational parental effects could have been responsible for the ameliorated response to heat seen in the progeny (Blödner *et al.*, 2007; Pecinka and Mittelsten Scheid, 2012). Consistent with this possibility, Suter and Widmer (2013*a*, 2013*b*) found that when heat stress was applied during vegetative growth only, phenotypic effects did not persist for more than one generation. Similarly, when Arabidopsis plants are infected with pathogens, increased resistance has been reported in the immediate progeny and in the second generation, but only when infections are carried out during the reproductive phase (Boyko *et al.*, 2010; Luna *et al.*, 2012; Slaughter *et al.*, 2012). Memory of salt stress across generations has also been investigated and the findings all point to an absence of *bona fide* transgenerational effects (Boyko *et al.*, 2010; Suter and Widmer, 2013*a*, 2013*b*; Groot *et al.*, 2016; Wibowo *et al.*, 2016). Similarly, a transgenerational effect of drought on the speed of germination has been reported by Ganguly *et al.* (2017), but in this case the effect diminished rapidly with each generation and is therefore unlikely to be responsible for a novel contribution to heritable variation. Furthermore, the experimental design did not allow for tests of transgenerational memory effects in plastic responses to drought, and an analysis of maternal variation and maternal trait-based effects was lacking.

It is now well established that DNA mutations are not the only source of heritable phenotypic variation in plants. An additional system of inheritance, often referred to as transgenerational epigenetics, typically involves stable differences in DNA methylation at or near transposable element (TE) sequences adjacent to genes (Quadrana and Colot, 2016). In the model plant Arabidopsis, most TE sequences are methylated at all cytosines, with methylation levels generally highest at CG sites (>80%), intermediate at CHG sites (40–60%), and lowest at CHH sites (<20%) (Cokus *et al.*, 2008; Lister *et al.*, 2008). TE sequences are methylated as a result of the combined activity of multiple DNA methyltransferases (Law and Jacobsen, 2010; Stroud et al., 2013, 2014) and can be actively demethylated by DNA glycosylases, which excise methylated cytosines from DNA (Law and Jacobsen, 2010). Demethylation is most pronounced in the central cell and leads to global hypomethylation of TE sequences in the endosperm, particularly on maternally derived chromosomes (Satyaki and Gehring, 2017). In contrast, because methylation dynamics in TE sequences occur almost exclusively at CHG and CHH sites (Bouyer *et al.*, 2017; Kawakatsu *et al.*, 2017; Lin *et al.*, 2017), most TE sequences, thanks to the CG sites they contain, remain highly methylated in the female and male germlines, as well as in the embryo. Thus, the limited reprogramming of methylation patterns between generations during normal development implies a considerable potential for genome-wide transgenerational epiallelic variation following accidental loss of DNA methylation. However, because the *de novo* DNA methylation machinery targets different TE sequences with varying efficiency (Teixeira *et al.*, 2009; Zemach *et al.*, 2013), this potential is not uniformly distributed among TE-containing alleles. Specifically, while experimentally induced epiallelic variation can persist for at least eight generations and presumably many more at some TE-containing loci, it is fully erased within one or a few generations at others (Johannes *et al.*, 2009; Teixeira *et al.*, 2009; Colome-Tatche *et al.*, 2012). Consequently, Arabidopsis accessions with different TE landscapes are expected to differ in their potential for transgenerational epigenetic variation. Finally, in the few cases where this has been examined, changes in DNA methylation were observed in response to stressors and some of these changes were transmitted, but transmission was again limited to the immediate progeny (Secco *et al.*, 2015; Wibowo *et al.*, 2016; Ganguly *et al.*, 2017).

Here, we set out to determine whether mild water deficit—a common stressor that plants face in natural settings and to which plastic responses have been demonstrated at phenotypic and gene-expression levels in Arabidopsis (Tisné *et al.*, 2013, Cubillos *et al.*, 2014)—could lead to new or altered intergenerational and transgenerational effects. We used a well-controlled multigenerational experimental design where the magnitude and timing of drought in the early part of the plant life cycle was replicated across generations for four natural accessions together with the reference accession Col-0. We phenotyped two generations in detail and used individual relative growth rates as integrated measures of stress response. We carried out a validation of gene-expression responses to drought using RNA-seq and an assessment of the likelihood of intergenerational epigenetics at the DNA methylation level in Col-0. Our results showed that mild drought induced phenotypic plasticity in each of the five accessions, but did not lead to any significant changes in terms of heritable effects. In addition, our DNA methylome data indicated that mild drought induced only intragenerational changes in DNA methylation, which were restricted to CHH sites and predominantly affected TE sequences. Taken together, our findings confirm that plants do not commonly generate transgenerational effects in response to changes in the environment, while intergenerational effects do occur.

## Material and methods

### Plant material and growth conditions

**Fig. 1.**
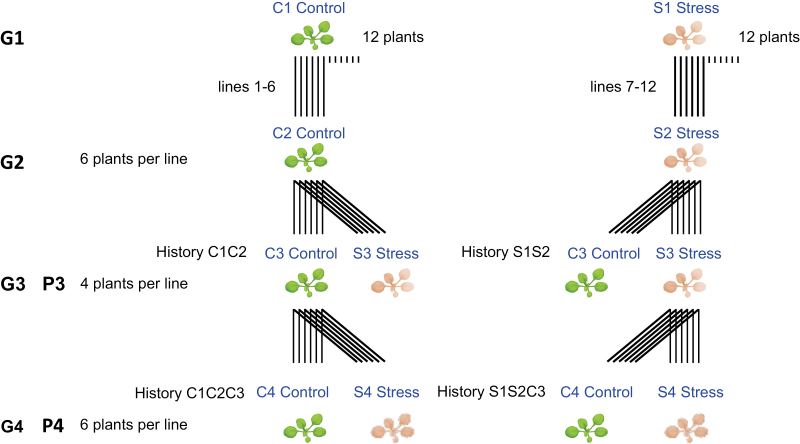
Schematic representation of the multigenerational experimental design. G, generation of growing plants; P, phenotyping experiment.

To investigate interactions between genotype and environment (G×E) in response to mild drought (Bouchabke *et al.*, 2008), we used the following accessions, which we obtained from the Versailles stock center (http://publiclines.versailles.inra.fr/): Col-0 (stock no. 186AV), Shahdara (Sha; 236AV), Bur-0 (172AV), Tsu-0 (91AV), and Cvi-0 (166AV). The four natural accessions were chosen because they show similar flowering time to Col-0 but have extensive divergence in DNA methylation among themselves and with Col-0 (Kawakatsu *et al.*, 2016a; The 1001 Genomes Consortium, 2016), presumably in large part as a result of numerous differences in their TE landscapes (Quadrana *et al.*, 2016; Stuart *et al.*, 2016). Isogenic lines for each accession were grown under well-watered control and mild drought-stress conditions for four generations (Fig. 1), using the Phenoscope robotic platform for high-throughput phenotyping (https://phenoscope.versailles.inra.fr/). This ensured uniform conditions during vegetative growth and enabled precise phenotype tracking (Tisné *et al.*, 2013). Control and mild drought stress conditions in each generation were chosen on the basis of the phenotypic responses observed by Bouchabke *et al.* (2008) and Tisné *et al.* (2013) over a range of drought levels, and on differences in gene expression found by Clauw *et al.* (2015) and Cubillos *et al.* (2014) under the same conditions that we employed in this current study. The growth conditions have been described in detail by Tisné *et al.* (2013). In the first generation (G1), 12 individuals (descended from the same mother plant) were grown per accession and per treatment. Half were used to establish six independent founder lines, which were maintained throughout the experiment by single-seed descent propagation. In the following generations, six replicates were grown per accession, treatment, and treatment history, with the exception of the third generation (G3) where only four replicates were grown due to space limitations on the Phenoscope (Fig. 1). Briefly, seeds were stratified for 4 d in the dark at 4 °C and germinated for 8 d on peat moss plugs before these were transferred to the Phenoscope. Individual plants were then cultivated for 21 d under short days (8-h photoperiod) to minimize developmental differences between accessions and to delay the flowering transition (Mockler *et al.*, 2003). During germination, the peat moss was saturated with water. During the first week on the Phenoscope (days 9–15 after sowing), the water content was gradually decreased through controlled watering until it reached either 60% (control, C) or 30% (stress, S), and these levels were then strictly maintained until day 29 after sowing. The plants were then moved to a standard growth chamber with optimal watering and long-day conditions to allow flowering and seed production. This strategy ensured that gametes or seeds were not themselves exposed to mild drought, thus minimizing intergenerational exposure effects. Seeds were collected from a single random individual per line×treatment history.

For the subsequent generations, seeds were collected and sieved to avoid sowing seeds that were in either the top or the bottom 10% of the size distribution of each line. The ranges of seed size varied between the accessions (in particular with Cvi-0 and Bur-0 having bigger seeds than Col-0 and Sha), but not between lines within accessions. Two phenotyping experiments were conducted (P3 and P4; Fig. 1) on lines from two treatment histories (P3: S1S2 versus C1C2; and P4: S1S2C3 versus C1C2C3).

### Phenotyping

Zenithal rosette images of each individual plant were taken daily and segmented as described previously (Tisné *et al.*, 2013) to extract the projected rosette area (PRA; a good proxy for rosette biomass at these developmental stages), rosette radius (the radius of the circle encompassing the rosette), compactness (the ratio between PRA and rosette circle area), together with the red, green, and blue components of the segmented rosette image. Here, we report our phenotypic analyses of the last two generations of plants grown on the Phenoscope (P3 and P4).

### Size and relative growth analysis

The data for each accession and generation were separately analysed in detail. The sample sizes differed between P3 and P4. In simplified models covering both generations, this made the P4 data weigh more on the results when parameters were shared between groups. We analysed all data jointly for hypothesis-testing on differences between generations and accessions.

Plant cohorts might contain groups with different properties and responses to treatments, while group membership might be unknown for each individual. To investigate such large initial heterogeneity among the plants selected for growth on the Phenoscope, the initial PRA distributions on day 9 after sowing (essentially the summed cotyledon areas) were inspected by finite mixture analysis (FlexMix library; Grun and Leisch, 2007). Gaussian models for initial log(PRA) with different numbers of component distributions (1–3) and different fixed treatment effects (stress/control in P3 or P4, stress/control in G1 and G2, i.e. G1/G2) for each component were compared using three information criteria (Akaike Information Criterion, AIC; Bayesian Information Criterion, BIC; and Integrated Complete Likelihood, ICL; Biernacki *et al.*, 2000). Models with single Gaussian components consistently had the lowest values of the information criteria. There were no indications of hidden large heterogeneities among the plants installed on the Phenoscope.

Initial and final values of log(PRA) were further studied in detail using linear mixed models (Pinheiro and Bates, 2000). The maximal models fitted contained random line effects with different variances in the G1/G2 control and drought groups. Models with more involved random effect specifications did not converge. Fixed effects were the exposure to drought in G1/G2 (intergenerational effect in the analysis of P3 and transgenerational effect in P4), treatment in P3 or P4 (plasticity), pot-order effects on the Phenoscope, and interactions of these variables. The maximal model contained heterogeneous error variances, different for each control/drought combination in G1/G2 and P3/P4. Model comparisons and simplifications were carried out using likelihood ratio tests (LRTs; using a restricted maximum-likelihood fit for random effects, maximum-likelihood for fixed). Non-significant effects were removed one by one. Simplifications were first attempted in the random effects, then in the error variances, and finally in the fixed effects, starting with the highest order interactions. Tests and estimates for random effects and error variances are reported for a restricted maximum-likelihood model containing all fixed effects. *P*-values for fixed effects are reported with respect to the minimum adequate model selected, i.e. the first model encountered that only contained significant effects.

We analysed relative growth rates of PRA in order to understand the gradual response to environmental conditions. We first inspected the per-day relative increase of log(PRA) using generalized additive mixed models (gamms; Wood, 2017) and subsequently fitted mixed linear models (Pinheiro and Bates, 2000) to the growth rates within age intervals where these appeared linear. For each treatment combination and accession, a gamm was fitted with smooths for day (age) and for pot-order effects (pot number). The gamms assume random variation between individuals and exponentially decaying correlations between observations on the same individual. The mixed linear models fitted to restricted age intervals contained the same variables as the models for final size above, with the addition of random variation between individuals within lines, fixed-age (day) effects, and interactions of age with the other fixed effects.

When testing for differences of effects between generations and accessions, we fitted all data jointly, with the random effects and error variances as above, but with different levels for generations and accessions added. In many cases this most-elaborate model failed to converge and we reduced the amount of random effect and error variances fitted until it did. We then carried out model simplification and hypothesis-testing on the fixed effects, which included interactions of the fixed effects above with generation and accession effects.

### Phenotypic trait-based maternal effects

Our detailed analysis of PRA accounted for effects of ancestral environments in G1/G2, but it did not include trait-based maternal effects (Kirkpatrick and Lande, 1989). These can lead to rapidly decaying or lasting transgenerational effects caused by individual parental variation. To investigate such effects and whether ancestral environments (i.e. memory) affected their strength and transmission, we analysed all traits recovered from the digital images in an equal manner. We restricted the analysis to the set of traits measured in both G2, P3, and P4, except for the image green component mean, which had a correlation coefficient with the red component greater than 0.9. For each accession, we thus fitted linear mixed-effects models to the log-transformed trait values after day 23. This part of the age trajectory of these traits was always approximately linear. We used individual maternal trait values on day 29 of G2 or G3 as explanatory variables to model the trait-based effects. For all traits, we tested whether trait-based maternal effects were present and if they differed between treatments in G1/G2 (ancestral environment×maternal trait interaction) and treatments in P3 or P4 (plasticity×maternal trait interaction). We removed data for a few plants with outlying patterns for the increase in log(PRA) before analysis: observations with a Cook’s distance value (in a simple regression on age) that was greater than 1 over the number of observations were removed. We fitted maximal mixed models to the data for each accession and for each trait with random effects of line and individual, and with heterogeneous error variances that could differ between environmental treatments experienced in G1/G2 and P3/P4. The model contained fixed effects of the G1/G2 and P3/P4 environmental treatments, pot-order effects, age effects, the effects of the maternal trait values (difference from the overall mean), and interactions of these (except for age×trait interactions and interactions between maternal traits). Model selection was conducted as above. However, we observed that selected models often had confidence intervals for maternal-effect slopes that still overlapped with zero or with each other, and we simplified such effects out of the models. To have a simple graphical means to assess the validity of mixed model predictions, we also fitted linear regressions to offspring trait–maternal trait combinations. All statistical analyses were conducted using R (www.r-project.org).

### RNA-seq

To confirm effects of mild drought on gene expression, we performed RNA-seq on leaves sampled from drought and control Col-0 plants (three replicates per treatment, six datasets). Leaf tissue was collected at 23 d after sowing from three Col-0 individuals grown on the Phenoscope under each of the control and mild-drought conditions and from the same seed batch as the founding Col-0 individuals used in the transgenerational design. Total RNA was extracted using a Qiagen RNAeasy extraction kit and sequenced at the Genome Center of the Max Planck Institute for Plant Breeding Research in Cologne, Germany. RNA-seq libraries were constructed using the standard Illumina Truseq protocol and sequenced in an Illumina Hiseq 2500 instrument. Between 18.3–23.7 million reads were obtained per sample (mean 20.7m) and aligned to the TAIR10 reference genome using TopHat2 with default parameters (Kim *et al.*, 2013). Reads aligning to multiple locations were removed using the samtools view with parameter -q 5 (Li *et al.*, 2009). After this filter, between 95.5–96.8% of the obtained reads were aligned to the reference genome. The number of reads per transcript was counted using the Bioconductor packages Rsamtools and ShortRead (Morgan *et al.*, 2009). Differential expression between samples in the control and drought conditions was calculated with the DEseq2 package in R (Love *et al.*, 2014). Genes with *q*-values <0.05 and log_2_FC >0.5 were considered as differentially expressed. TE differential expression was analysed using TEtools (Lerat *et al.*, 2017). We used the Panther classification system for Gene Ontology (GO) analysis (Mi *et al.*, 2019) and used Fisher’s exact tests with Benjamini–Hochberg false discovery rate (FDR) corrections to test for over-representation of functional classes corresponding to different biological processes. Only the results for genes with FDR<0.05 were inspected.

### Whole‐genome bisulfite sequencing

To investigate plasticity of genomic DNA methylation patterns in response to mild drought, whole‐genome bisulfite sequencing (WGB-seq) was performed on pooled DNA extracted at day 29 after sowing from mature leaves of 12 Col-0 plants from the control and water stress treatments (one pooled sample per treatment, two datasets). To assess intergenerational effects, we performed independent WGB-seq experiments on 10-d-old seedlings from five independent C1C2 and five independent S1S2 G2 lines grown on the Phenoscope under standard *in vitro* conditions (10 datasets). MethylC-seq library preparation and sequencing was performed by BGI (Shenzhen, China) using standard Illumina protocols. Adapter and low-quality sequences were trimmed using Trimming Galore v.0.3.3. Mapping was performed on the TAIR10 genome annotation using Bismark v.0.14.2 (Krueger and Andrews, 2011) with the following parameters: --bowtie2, -N 1, -p 3 (alignment); --ignore 5 --ignore_r2 5 --ignore_3prime_r2 1 (methylation extractor). Only uniquely mapping reads were retained. The methylKit package v.0.9.4 (Akalin *et al.*, 2012) was used to calculate individual differentially methylated positions (DMPs), and differentially methylated regions (DMRs) in 100-bp non-overlapping windows. The significance of calculated differences was determined using Fisher’s exact tests and Benjamin–Hochberg (BH) adjustment of *P*-values (FDR<0.05) and methylation difference cut-offs of 40% for CG, 20% for CHG, and 20% for CHH. Differentially methylated windows within 100 bp of each other were merged to form larger DMRs. Cytosine positions covered by more than 100 reads were not considered. For DMP analysis only cytosines covered by a minimum of six (CG and CHG) and 10 (CHH) reads in all libraries were considered. Bisulfite conversion rates were estimated by the number of methylated cytosine calls in the chloroplast genome.

The RNA-seq and MethylC-seq sequencing data have been deposited in the ENA short-read archive (https://www.ebi.ac.uk/ena; project no. PRJEB27682).

## Results

### Immediate phenotypic plasticity and gene expression changes in response to mild drought

**Fig. 2.**
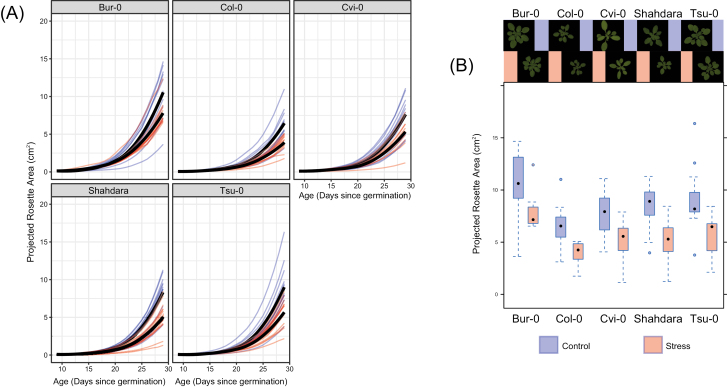
Descriptive analysis of growth curves for the projected rosette area (PRA) in the first generation (G1) for the five accessions grown under control or mild drought conditions. Individuals where PRA remained below 1 cm^2^ by the end of the experiment or that died prematurely are not shown. (A) Kinetics of shoot size estimated by daily measurements of PRA. Growth curves of plants in control conditions are in blue, those that experienced mild drought are in red. Black lines represent the means for each group. (B) Box-and-whiskers plots showing PRA on day 29 for the plants in each accession×treatment combination. Representative images of the plants are shown. See Fig. 1 for experimental design.

As expected (Tisné *et al.*, 2013), the growth dynamics of the projected rosette area (PRA) showed clear phenotypic plasticity in response to mild drought for each accession analysed and in each generation where the stressor was imposed. PRA decreased significantly when accessions were grown under mild drought (in generation G1; Fig. 2A): depending on the accession, at 29 d after sowing PRA values were 27–40% lower than in control conditions (G1; Fig. 2B). All accessions therefore had reduced growth in drought conditions, which is evidence of stress (Claeys *et al.*, 2014). Consistent with results of previous studies of the transcriptional response to mild drought (Cubillos *et al.*, 2014; Clauw *et al.*, 2015), differential analysis of the two RNA-seq datasets identified significant changes in steady state mRNA levels for 468 genes (FDR<0.05, log_2_FC>0.5; 205 and 263 genes with lower and higher expression under mild drought, respectively; Supplementary Table S9 at *JXB* online), but not for any of the annotated TE sequences. A possibility is that the mild drought was too weak as a stressor to overcome the typically strong epigenetic silencing of TE sequences. GO analysis indicated over-representation of several stress-related categories, including ‘cell wall thickening’ and ‘responses to water deprivation’ (Supplementary Table S10).

### Intergenerational and transgenerational environmental effects

We then compared, in as much detail as possible, the phenotypic traits of plants whose parents experienced drought treatments for two consecutive generations and the traits of their progeny (Fig. 1). Phenotyping at P3 compared C1C2 versus S1S2 and assessed intergenerational effects, whilst phenotyping P4 compared the C1C2C3 versus S1S2C3 treatment histories. The G3 generation without drought treatment allowed only transgenerational effects to contribute to the effects of treatment history in P4.

The effects of drought in G1/G2 on initial log(PRA) differed between accessions and generations (*χ*^2^_4_=11.9, *P*=0.018), with Bur-0 and Sha being the most similar in their responses (Table 1). There were intergenerational effects but no transgenerational effects of drought on initial log(PRA), and the intergenerational effects differed between the accessions. With regards to final log(PRA), we could not demonstrate any significant interactions between factors and generation or accession effects when analysing all the data together. We found significant overall plasticity (*χ*^2^_1_=92.8, *P*<0.0001), in agreement with the results for G1 (Fig. 2), and a trend for an overall effect of drought in G1/G2 (*χ*^2^_1_=3.50, *P*=0.06). When analysing accessions and P3 and P4 separately, we again detected intergenerational effects on initial log(PRA) and also on final log(PRA). Plants from a history with drought were smaller (Table 1). In this case, we did not find any transgenerational effects of exposure to drought on initial and final log(PRA) of P4. The trend for an overall effect of drought on final size in the analysis with P3 and P4 combined must therefore be ascribed to the intergenerational effects that were detected, and not to any transgenerational effects. Unexpectedly, we sometimes found effects of drought on initial log(PRA) heterogeneity (residual variance). These effects must have been spurious, as the stress treatment had not yet started at this stage. Therefore, whilst we modeled variance heterogeneity throughout, we do not present or interpret the results. For mean final size, we found that all accessions except Sha showed significant plasticity in each generation. Sizes were larger in the control treatment (Table 1).

**Table 1.** Effects of environmental states in G1/G2 and phenotypic plasticity on the initial and final mean projected rosette area [log(PRA)] in generations P3 and P4

Accession	Intergenerational effect of drought in G1/G2	LRT	Plasticity P3 (Control–Drought)	LRT	Transgenerational effect of drought in G1/G2	LRT	Plasticity P4 (Control–Drought)	LRT
Size on day 8 after germination								
Col-0	[–0.143, –0.016]	*P*=0.013	–	NS	–	NS	–	NS
Bur-0	[– 0.414, –0.129]	*P*=0.001	–	NS	–	NS	–	NS
Cvi-0*	[–0.128, –0.035]	*P*=0.001	–	NS	–	NS	–	NS
Tsu-0	NS	NS	–	NS	–	NS	–	NS
Sha	[–0.322, –0.167]	*P*<0.001	–	NS	–	NS	–	NS
Size on day 29 after germination								
Col-0	–	NS	[0.175, 0.318]	*P*<0.001	–	NS	[0.197 – 0.375]	*P*<0.001
Bur-0	[–0.211, –0.092]	*P*<0.001	[0.330, 0.448]	*P*<0.001	–	NS	[0.317 – 0.546]	*P*<0.001
Cvi-0*	–	NS	[0.397, 0.509]	*P*<0.001	–	NS	[0.394 – 0.557]	*P*<0.001
Tsu-0	–	NS	[0.305, 0.423]	*P*<0.001	–	NS	[0.250 – 0.384]	*P*<0.001
Sha	[–0.228, –0.026]	*P*=0.013	[0.310, 0.449]	*P*<0.001	–	NS		NS

Confidence interval results are shown from linear mixed models for each accession, based on parameter estimates of minimum adequate models; * indicates an accession where the variance between lines is retained in the model. All tail probabilities are from *χ*^2^_1_ tests. NS, not significant; LRT, likelihood ratio test; Sha, Shahdara.

**Fig. 3.**
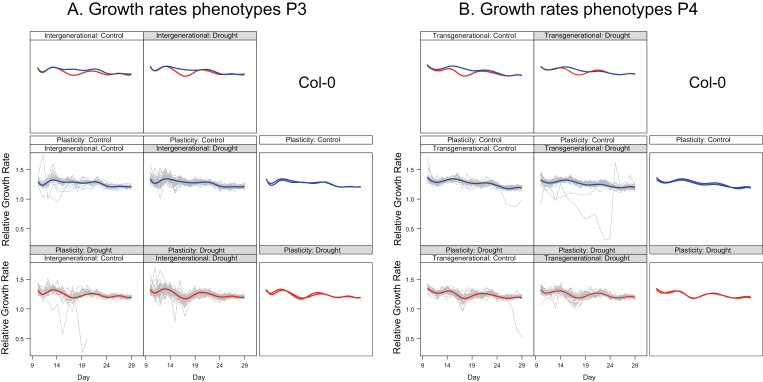
The time-dependent pattern of relative growth rates in P3 and P4 as predicted for the first pot on the Phenoscope (the model accounts for pot order) for accession Col-0, based on generalized additive mixed models (gamms). The age of the plants in days is given on the bottom axis: recording started at 9 d after sowing. The panels with grey lines show the raw data for each treatment combination, together with the predicted trajectories of relative growth rate for each combination of treatments in G1/G2 (Memory) and P3 or P4 (Plasticity). P3/P4 treatments are shown in blue (control) and red (drought). The same predicted trajectories are shown in the panels above and to the right, so that pairwise comparisons can easily be made between the ancestral drought treatment and drought in the phenotyping generation (plasticity). The graphs indicate clear growth plasticity in response to mild drought and that plants managed to compensate for the initial drop in relative growth rate shortly after the mild drought had reached a stable level at day 20. Note that there are very few plants with outlying patterns, and that they have very low growth rates only within a restricted age window. See Fig. 1 for experimental design.

Our gamm analyses indicated that relative growth rates (RGRs) from days 13–16 and from days 25–28 could be considered linear for each individual (Fig. 3, which shows representative results for Col-0 in P3 and P4). For early RGR, we could not demonstrate differences between accessions in plasticity or effects of drought history. However, there were significant differences in plasticity and drought effects between P3 and P4 (day×plasticity×generation: *χ*^2^_1_=4.87, *P*=0.03; day×drought in G1/G2×generation: *χ*^2^_1_=10.9, *P*=0.001). For late RGR, we found a weakly significant effect of drought history (*χ*^2^_1_=3.96, *P*=0.05). Plasticity in late RGR differed between accessions (accession×plasticity *χ*^2^_1_=10.16, *P*=0.04) and between generations (day×generation×plasticity *χ*^2^_1_=12.2, *P*=0.0004). In the analyses for each generation and accession separately, we did not detect transgenerational effects but did find significant intergenerational effects of drought on RGR from days 13 to 16 in three accessions and in a single accession from days 25–28 (Tables 2, 3, Fig. 3). Plants from a history with drought grew relatively more: this could be seen as evidence of a stress memory. Comparing plastic responses for RGR from days 13 to 16, these were found to be identical in generations P3 and P4 (Table 2). Modeling RGR revealed a steeper decrease with age after the reduction in water supply in all the accessions (Table 2, Fig. 3). In control conditions on day 13, plants grew equally to or faster than those subjected to mild drought, and while the RGR of plants under drought declined with age, in control conditions the decline was much less or was absent. The plastic phenotypic responses in P3 showed the same qualitative pattern for days 25–28 as for earlier RGRs. However, for three out of five accessions in P4, plants subjected to mild drought acclimated and their RGR recovered to a similar level to that of control plants (days 25–28; Table 3, Fig. 3). For Cvi-0 we observed the same pattern as in P3. We found compensatory growth for Shahdara: RGR decreased less with age in the drought group and on day 28 the RGR of this group was faster than for the control. Hence the strength of RGR plasticity could apparently vary between generations, potentially dependent on environmental history, but not at all plant ages. There were no transgenerational effects of exposure to drought in G1/G2 on mean growth rate values in P4. Different magnitudes of between-individual variance across the drought histories did occur for Col-0, Cvi-0, and Bur-0. Individual variation in RGR was larger from days 13–16 for the Col-0 plants in P4 that descended from parents in the G1/G2 drought group (*P*<0.001); for Bur-0, it was larger in P3 (*P*=0.045) and smaller in P4 (*P*=0.022). For Col-0, the growth rate variance from days 25–28 was smaller among descendants of the G1/G2 drought group (*P*<0.001). For Cvi-0, this variance was larger in P3 and P4 (*P*=0.027, *P*<0.001). This pattern indicated that the amounts of trait variation at the end of an experiment or at the time of measurement could be determined by intricate time-dependent variances in growth processes. The gene expression responses in the plants could thus overcome the stress, but this was not uniformly the case.

**Table 2.** Effects of environmental states in generations G1/G2 and P4 on the mean relative growth rate from days 13–16 in P3 and P4

Accession	Generation	Drought in G2 effect on intercept	Drought in G2 × Day slope	LRT	Plasticity (intercept difference between Control–Drought groups)	Plasticity × Day slope	LRT
Col-0	P3 (inter-)	[–0.006, 0.047]	[–0.026, –0.000]	*P*=0.042	[–0.032, 0.025]	Drought [–0.052, –0.026]; Control–Drought [0.030, 0.059]	*P*<0.001
Col-0**	P4 (trans-)			NS	[–0.003, 0.026]	Drought [–0.052, –0.041]; Control–Drought [0.033,0.046]	*P*<0.001
Bur-0**	P3 (inter-)	[0.010, 0.029]	–	*P*<0.001	[–0.011, 0.017]	Drought [–0.073, –0.060]; Control–Drought [0.038, 0.053]	*P*<0.001
Bur-0** ^,†^	P4 (trans-)			NS	[–0.020, 0.016]	Drought [–0.073, –0.060]; Control–Drought [0.045, 0.060]	*P*<0.001
Cvi-0	P3 (inter-)			NS	[0.002, 0.045]	Drought [–0.077, –0.059]; Control–Drought [0.024, 0.046]	*P*<0.001
Cvi-0	P4 (trans-)			NS	[0.007, 0.061]	Drought [–0.058, –0.037]; Control–Drought [0.017, 0.046]	*P*<0.001
Tsu-0*	P3 (inter-)			NS	[–0.008, 0.034]	Drought [–0.051, –0.036]; Control–Drought [0.025, 0.044]	*P*<0.001
Tsu-0*	P4 (trans-)			NS	[–0.009, 0.024]	Drought [–0.062, –0.049]; Control—Drought [0.033, 0.049]	*P*<0.001
Sha	P3 (inter-)	[0.007, 0.020]		*P*<0.001	[–0.037, 0.015]	Drought [–0.094, –0.067]; Control—Drought [0.057, 0.085]	*P*<0.001
Sha*	P4 (trans-)			NS	[–0.075, –0.000]	Drought [–0.107, –0.080]; Control—Drought [0.077, 0.106]	*P*<0.001

Results are from linear mixed model analysis and models after selection. Age in number of days is rescaled to value zero at day 13, to help the interpretability of estimates. ** Accessions with random effects that differ in line/individual variance between the two G1/G2 treatment levels. * Accessions where the variance between lines is retained in the model. ^†^ Accessions for which the pot effects (linear effect of pot number on the Phenoscope) were retained. NS, not significant; LRT, likelihood ratio test; Sha, Shahdara.

**Table 3.** Effects of environmental states in G1/G2 and P4 on the mean relative growth rate from days 25–28 in P3 and P4

Accession	Generation	Drought in G2 effect on intercept	Drought in G2 × Day slope	LRT	Plasticity (intercept difference between Control–Drought groups)	Plasticity × Day slopes	LRT
Col-0	P3 (inter-)				[–0.001, 0.024]	Drought [–0.009, –0.003]; Control–Drought [0.001, 0.010]	*P*=0.011
Col-0** ^,†^	P4 (trans-)			NS			NS
Bur-0	P3 (inter-)	[0.002, 0.012]	NA	*P*=0.006	[0.020, 0.044]	Drought [–0.025, –0.019]; Control–Drought [0.006, 0.015]	*P*=0.011
Bur-0*	P4 (trans-)			NS			NS
Cvi-0** ^,†^	P3 (inter-)			NS	[0.023, 0.048]	Drought [–0.011, – 0.006]; Control–Drought [0.004, 0.012]	*P*<0.001
Cvi-0**	P4 (trans-)			NS	[0.019, 0.045]	Drought [–0.020, –0.015]; Control–Drought [0.007, 0.015]	*P*<0.001
Tsu-0^,†^	P3 (inter-)			NS	[0.028, 0.055]	Drought [–0.030, –0.022]; Control–Drought [0.009, 0.018]	*P*<0.001
Tsu-0* ^,†^	P4 (trans-)			NS			NS
Sha	P3 (inter-)			NS			NS
Sha	P4 (trans-)			NS	[–0.053, – 0.018]	Drought [–0.003, –0.002]; Control–Drought [–0.014,– 0.001]	*P*=0.021

Results are from linear mixed model analysis. Age in number of days is rescaled to value zero at day 28, to help the interpretability of estimates. ** Accessions with random effects that differ in line/individual variance between G1/G2 groups. * Accessions where the variance between lines is retained in the model. ^†^ Accessions for which the pot effects (linear effect of pot number on the Phenoscope) were retained. NS, not significant; LRT, likelihood ratio test; Sha, Shahdara.

### Limited presence and persistence of maternal trait-based effects

**Fig. 4.**
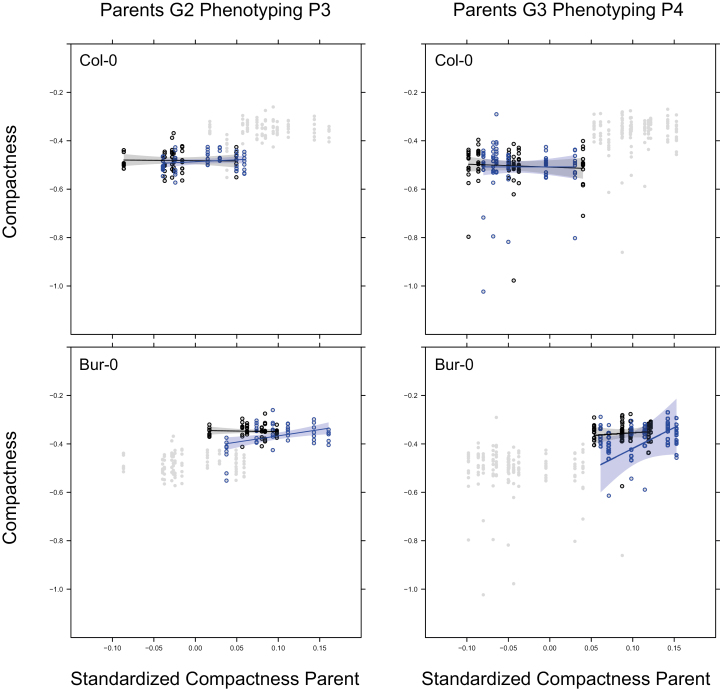
Effects of maternal traits in Col-0 and Bur-0 for P3 and P4. The data points of both accessions are shown in grey for each phenotyping experiment. Dependencies of the offspring trait values on maternal trait values are shown for log-transformed rosette compactness (see Methods). Linear regressions are estimated for each ancestral drought environment. Data points are circled in blue for individuals with ancestors under drought in G1/G2, and circled in black for individuals with ancestors under control conditions. See Fig. 1 for experimental design.

In 11 out of 250 tests of models with maternal trait effects on individual phenotypes in P3 or P4 the slope of the maternal trait regression was found to depend on the environmental regime experienced by the ancestors in G1/G2 (Supplementary Tables S1, S2). In two cases it depended on the environmental regime in P4. By inspecting the models for each accession, we were able to make an assessment of whether these changes in the maternal trait slopes would affect the persistence of the maternal effects. This could be the case when the effects of traits on themselves become larger in absolute value or when a causal chain of traits carries a stronger weight (Kirkpatrick and Lande, 1989). Compactness and mean blue value were the only maternal trait-based effects where we found that the trait had an effect on itself. There were no enchained maternal trait effects that could lead to lagged responses. When we inspected the maternal trait dependency of compactness in Col-0 and Bur-0 (Fig. 4), we observed that the ranges of the maternal trait values differed between descendants of stress and control treatments in G1/G2.

The results did show that the maternal trait models had slopes that could depend on historical and current environments, and that the scope for a change in the persistence of maternal effects due to mild drought was limited (Fig. 4). The slopes of the effects were not particularly strong or general across accessions, and they did not seem to be a valid candidate for persistent transmission of trait variation. The fraction of tests significant for effects of maternal traits on offspring and with interpretable confidence intervals was very close to a 5% type-I error rate (13/250). We therefore concluded that the number of heritable effects transmitted through maternal trait-based effects was negligible.

In 18 out of 50 trait×accession models, individual variation was increased in the drought environment in P3 or P4 (Supplementary Tables S3, S4). In 10 out of 50 models, the variation between individuals was larger among descendants of individuals descended from drought in G1/G2. In five cases this variance was smaller.

### Mild drought induces changes in DNA methylation unrelated to known transcriptome changes associated with drought stress

To complement the phenotypic analysis, we investigated the impact of mild drought on genomic DNA methylation patterns using the reference accession Col-0, which showed the strongest phenotypic response (Fig. 2B) and for which a wealth of epigenomic data are available. To investigate within-generation plastic responses and capture as many cumulative changes as possible, WGB-seq was performed on DNA extracted at day 29 from pooled leaves of treated and control plants at P4 (C1C2C3 descendants; Fig. 1; Supplementary Tables S5, S6). Overall, cytosine methylation levels were similar between control and stressed leaves (Supplementary Fig. S1a), although they were slightly higher than in previous reports (8.6% and 9% of methylated cytosines versus 6.7%; Cokus *et al.*, 2008; Lister *et al.*, 2008), presumably because of differences in mapping and methylation calling methods as well as in the organs examined. Other global measures, such as the distribution of methylation between the three types of sites and annotations, were also identical for control and stressed leaves (Supplementary Fig. S1b, c). We therefore concluded that mild drought did not directly affect overall DNA methylation patterns in Arabidopsis.

**Fig. 5.**
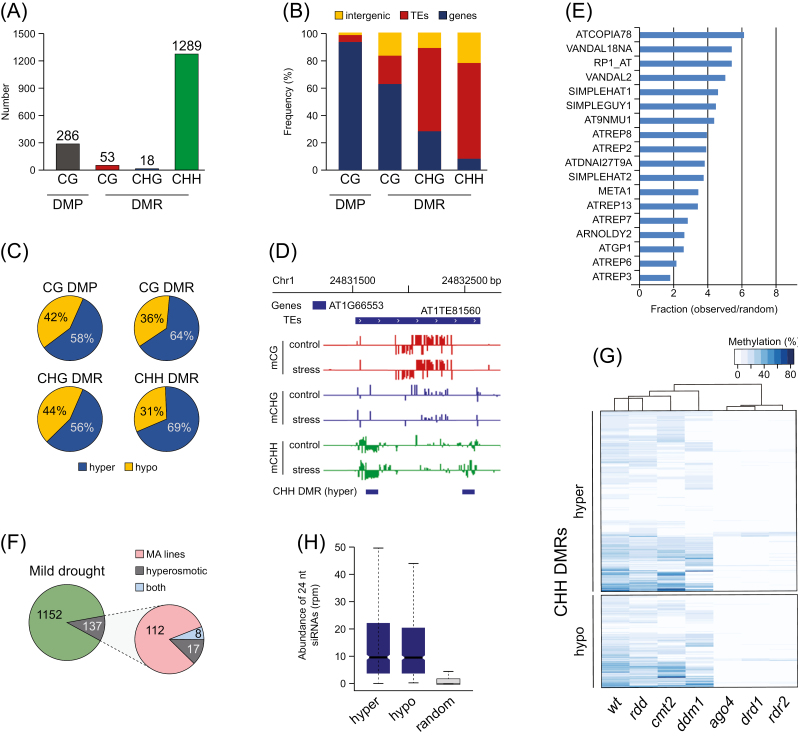
Characterization of stress-induced local changes in DNA methylation. (A) Number of differentially methylated positions (DMPs) and differentially methylated regions (DMRs) for each of the three types of site (CG, CHG, and CHH). (B) Annotation of DMPs and DMRs in relation to genes, transposable elements (TEs), and intergenic regions. (C) Distribution of local gains and losses of DNA methylation across DMPs and DMRs. (D) Example of CHH-DMRs on a TE. (E) Graphical representation of the 18 TE families that showed more DMRs than the random expectation (*P*<0.01). (F) Overlap (including 500-bp flanking windows) of DMRs induced by mild drought and DMRs found in mutation accumulation (MA) lines according to Becker *et al.* (2011) and Schmitz *et al.* (2011), and DMRs induced by hyperosmotic stress according to Wibowo *et al.* (2016) (G) Hierarchical clustering based on mean CHH methylation levels in wild-type (wt) and mutants for the RdDM (*rdr2*, *ago4*, and *drd1*), CMT2 (*ddm1* and *cmt2*), and DNA demethylation (*rdd*) pathways in regions overlapping hypo or hypermethylated CHH-DMRs according to Stroud *et al.*, (2013). (H) Abundance of 24-nt siRNAs in random TEs or those with hypo or hypermethylated CHH-DMRs.

To identify local differences, methylation levels were compared at individual cytosine positions as well as in 100-bp windows separately for each of the three types of sites (CG, CHG, and CHH; see Methods). Based on this approach, we were able to identify 286 differentially methylated positions (DMPs) and 1360 differentially methylated regions (DMRs), most of which were defined by single 100-bp windows (Supplementary Tables S7, S8). All DMPs mapped to CG sites whereas most DMRs (95%) mapped only to CHH regions (Fig. 5A). The vast majority of CG DMPs (93%) were within methylated gene bodies (Fig. 5A, B) and they reflected almost equally either increased or decreased methylation levels in treated plants compared to controls (Supplementary Fig. S2), consistent with the notion that gene-body methylation tends to vary stochastically across generations at individual CG sites (Becker *et al.*, 2011; Schmitz *et al.*, 2011; Jiang *et al.*, 2014). On the other hand, CHH-DMRs were mainly located over TE sequences and tended to reflect hypermethylation in plants experiencing drought (Fig. 5C, D, Supplementary Fig. S2).

As different TE families may show different sensitivities to environmental cues (Pecinka *et al.*, 2010;Yu *et al.*, 2013; Grandbastien, 2015; Matsunaga *et al.*, 2015; Quadrana *et al.*, 2016), we assessed whether CHH-DMRs were preferentially localized over specific TE families. Out of the 326 TE and other repeat families annotated in the TAIR10 Arabidopsis genome, 164 showed at least one DMR and 18 families were enriched in DMRs compared to the random expectation (Fig. 5E). These included the LTR-retrotransposon family *ATCOPIA78*, which is known to be sensitive to biotic and abiotic stress (Yu *et al.*, 2013; Matsunaga *et al.*, 2015; Quadrana *et al.*, 2016). On the other hand, only a small percentage of CHH-DMRs caused by mild drought overlapped with DMRs that arose spontaneously in mutation accumulation lines (9.3%; Hagmann *et al.*, 2015) or that were induced by hyperosmotic stress (1.9%; Wibowo *et al.*, 2016; Fig. 5F). Thus, we concluded that mild drought induced a limited number of robust DNA methylation changes over regions that were distinct from those subjected to stochastic or salt-induced DNA variation in methylation.

To further investigate the CHH-DMRs induced by mild drought, we compared their CHH methylation levels in different DNA methylation mutants as determined by Stroud *et al.* (2013) and found that most corresponded to regions targeted by the RNA-directed DNA methylation (RdDM) pathway (Fig. 5G), which involves the DNA methyltransferase DRM2 rather than by the alternative CHH maintenance methylation pathway mediated by the DNA methyltransferase CMT2. Consistent with these findings, TE sequences that overlapped drought-induced CHH-DMRs had a high abundance of matching 24-nt small RNAs (Fig. 5H). Moreover, no association was detected between drought-induced CHH-DMRs and regions subjected to active DNA demethylation (*rdd* mutant; Fig. 5G). We therefore concluded that mild drought mainly directly affected sequences targeted by RdDM.

None of the genes known to be involved in DNA (de)methylation appeared to be affected by mild drought, leaving the question open as to which factors induce DNA methylation changes during such conditions.

Among the 468 genes detected as being transcriptionally responsive to mild drought in our conditions (G1), only two were affected by CG DMPs, and were probably inconsequential given the lack of function associated with gene-body methylation. Another two genes were located less than 500 bp from a DMR (Supplementary Fig. S3a). These two DMRs were of the CHH type but did not correspond to annotated TE sequences. One DMR mapped to the promoter region of *AT5G35735*, which encodes an auxin responsive protein of unknown function. The other DMR was located within the first intron of *AT3G10340*, which encodes a putative phenylalanine ammonia-lyase that may be involved in plant defense against biotic and abiotic stresses (Raes *et al.*, 2003). Given the large size of the first intron (1.3 kb), it probably contained regulatory sequences (Morello and Breviario, 2008). Moreover, hypermethylation of the promoter DMR of *AT5G35735* and hypomethylation of the intronic DMR of *AT3G10340* in response to mild drought were associated with down- and up-regulation, respectively (Supplementary Fig. S3b). Taken together, these findings suggested a causal link between altered gene expression and altered DNA methylation for these two genes. However, the observation that most genes affected by mild drought were not proximal to drought-induced DMPs or DMRs indicated that plastic changes in DNA methylation had a marginal role in the phenotypic responses of the plants to mild drought. An alternative explanation for our results is that transcriptional and methylation patterns might be so heterogeneous and dynamic that only analyses of both on the same individuals and very close in time would be able to determine correlations. If this is the case, then it would clearly indicate the limited scope for propagation of effects.

### No intergenerational effects of mild drought on DNA methylation

Finally, we tested whether intergenerational DNA methylation changes occurred. Taking into consideration the possibility of cumulative effects over successive generations of growth under drought, G3 progenies of Col-0 C1C2 and S1S2 plants were chosen for further analysis (Fig. 1A). WGB-seq was performed on DNA extracted from unstressed individuals of the progeny of five Col-0 C1C2 and S1S2 founder lines (see Methods; Supplementary Tables S5, S6). Differential DNA methylation was examined as described above using the five C1C2 and S1S2 progenies as biological replicates. Following this approach, no single consistent DMR could be identified between the two types of progeny, and hence no intergenerational effect could be identified. In addition, we carried out all possible inter-individual comparisons, which again yielded no single DMR. However, there was a marginal increase in the amount of stochastic variation in DNA methylation for the three types of sites among progenies derived from the five stressed parental lines (Supplementary Fig. S4). Our results therefore suggested that there were no targeted and specific DNA methylation changes induced by mild drought that would persist into the next generation, although exposure to drought may have increased the heterogeneity of the methylome among progeny of stressed plants, and other intergenerational responses might still have left a later trace in the methylome.

## Discussion

It has been proposed that exposure to environmental cues can trigger phenotypic changes that become inherited for more than one generation, and that this occurs through epigenetic mechanisms (Bossdorf *et al.*, 2008; Richards *et al.*, 2017). In this study, we have shown that water deficit applied before the reproductive stage in two successive generations negatively affected the vegetative growth of individuals. As expected, we identified expression changes in genes involved in, for example, cell wall thickening and responses to water deprivation (Supplementary Table S10). However, mild drought did not affect overall DNA methylation patterns (Supplementary Tables S7, S8) and most genes with differential expression were not proximal to drought-induced DMRs (Supplementary Fig. S3a), which suggested a marginal role of changes in DNA methylation in the phenotypic response of plants to mild drought. We also detected intergenerational drought effects on the rosette sizes at the age at which the first measurements were made on the Phenoscope (Fig. 2) and effects on relative growth rates (Fig. 3). Transgenerational effects of mild drought after two successive generations of exposure were limited to changes in the amounts of individual variation in phenotypic traits (Table 3, Supplementary Table S4). The variance could increase or decrease depending on the accession and trait considered. Furthermore, the amount of stochastic variation in DNA methylation increased marginally in lines with ancestral drought (Supplementary Fig. S4), suggesting that intergenerational effects on variability can occur that might persist into the next generation, but without any clear directional phenotypic effects. Finally, we found only a limited number of maternal trait-based effects in any of the accessions (Supplementary Table S2, Fig. 4), and their overall occurrence was close to the type-I error rate. Our results therefore add to the growing body of evidence against transgenerational epigenetic changes being a predictable and common response of plants to changes in the environment (Boyko *et al.*, 2010; Suter and Widmer, 2013*a*, 2013*b*; Groot *et al.*, 2016; Wibowo *et al.*, 2016; Ganguly *et al.*, 2017).

### Intergenerational plasticity is limited and does not lead to transgenerational effects

Stressors consistently affect the expression of a large number of genes and in a number of cases they induce CHH hyper- or hypomethylation of a variable number of TE and other repeat sequences (e.g. Dowen *et al.*, 2012; Eichten and Springer, 2015; Secco *et al.*, 2015; Wibowo *et al.*, 2016; Fig. 5, Supplementary Tables S7, S8, S9). However, gene expression is rarely associated with changes in DNA methylation (Meng *et al.*, 2016) and for a given stressor, the extent of such changes as well as the mechanisms involved may differ radically between different species (Secco *et al.*, 2015). While many of the CHH-DMRs induced by salt stress in Arabidopsis are transmitted to the immediate progeny (Wibowo *et al.*, 2016), this was not the case for the CHH-DMRs induced by mild drought (Supplementary Figs S1, S3). There is also no transmission of the CHH-DMRs induced by phosphate starvation in rice (Secco *et al.*, 2015). Thus, the evidence so far points to a clear effect of environmental factors in triggering changes in DNA methylation; however, these do not persist in the offspring. Indeed, different mechanisms that prevent the transmission of environmentally induced epigenetic states across generations have been described (Baubec *et al.*, 2014; Crevillén *et al.*, 2014; Iwasaki and Paszkowski, 2014), and our design aimed to minimize intergenerational plasticity (Fig. 1). Nonetheless, true transgenerational epigenetic variation exists in nature (Silveira *et al.*, 2013; Quadrana and Colot, 2016) and what generates it remains unresolved. Analyses of natural populations are now just beginning to investigate this question, with no clear answers so far, except that most DNA methylation variants seen in nature are probably caused by DNA sequence variation and are therefore by definition not truly epigenetic (Durand *et al.*, 2012; Schmitz *et al.*, 2013; Li *et al.*, 2014; Dubin *et al.*, 2015; Kawakatsu *et al.*, 2016b; Niederhuth *et al.*, 2016; Quadrana *et al.*, 2016; Agorio *et al.*, 2017).

At the morphological level, we detected different responses between accessions, with both plasticity, intergenerational effects, and limited trait-based maternal effects playing a role. Maternal trait-based effects with potentially lasting effects occurred at low frequency (Fig. 4, Supplementary Table S2). Only for rosette compactness in a single accession (Bur-0) did an effect of a trait on itself occur in both P3 and P4; however, there was only an effect of ancestral drought on the slope in P3 and not in P4. Mild drought changed trait variances but did not consistently change the slopes of maternal trait-based effects (Fig. 4, Supplementary Tables S1–S4). Mild drought therefore did not change this presumed mechanism of non-genetic heritability.

### Plasticity is probably adaptive, a memory effect is probably not

Modeling suggests a potential for transgenerational epigenetics based on DNA methylation to endow plants with a means to generate adaptive heritable phenotypic variation in response to changing environments (Bossdorf *et al.*, 2008; Geoghegan and Spencer, 2013*a*, 2013*b*; Uller *et al.*, 2015; Kronholm and Collins, 2016). Models have shown that environmentally induced epiallelic variation can be favored over purely stochastic switching (Furrow and Feldman, 2014). We did not detect such phenotypic changes and did not even find specific intergenerational changes in methylation. The general lack of such effects in our experiment might indicate that they are actually unnecessary within the range of environments that we imposed. In agreement with models of adaptation (Kuijper and Hoyle, 2015), we found that responses by means of phenotypic plasticity were stronger than those by maternal effects. The rate at which intergenerational drought effects rapidly decreased with plant age in the P3 group (Fig 3, Tables 1–3) and the absence of transgenerational effects (Fig 4,Tables 1–3) seems to indicate that mild drought stress did not induce strong physiological changes that persist for very long and that would affect transmission of information or resources. On the contrary, a number of days after drought set in, several accessions in P4 managed to return to the same relative growth rates as in our control (Fig 4, Table 3), demonstrating acclimation, and in one accession over-compensation. This suggests that there was little remaining stress at that point. However, we did not find the same pattern in P3. Further experiments that include additional accessions with incomplete recovery would allow testing of whether lines with persistently incomplete acclimation have more transgenerational effects and whether ancestral environmental history effects could indeed explain this variability in the strength of plasticity. The adaptiveness of stress responses in certain conditions could be investigated further using Finlay–Wilkinson regressions (Finlay and Wilkinson, 1963). This would require sampling seeds for each individual in the experiment in order to have lifetime measures of fitness or yield, plus additional levels of (mild) drought to permit fitting of regressions.

The intergenerational responses that we observed could be consequences of environmental effects on seed investment. The changes in trait variance could indicate that mild drought stress affects how well plants are able to predict the near future, without much of a consistent trend towards an improved or decreased predictability. The changes that we observed might then be more in support of a bet-hedging strategy (e.g. Crean and Marshall, 2009).

### Conclusions

Our study provides strong support for the notion that plants first respond to physiological stressors through well-defined and conserved transcriptional networks (Juenger, 2013; Ding *et al.*, 2014; Clauw *et al.*, 2015) or immediate parental influences on offspring phenotypes (Herman and Sultan, 2011; Wibowo *et al.*, 2016). It remains to be determined whether transgenerational epigenetic variation in nature is caused by more dramatic environmental conditions than those tested so far in the laboratory, by combinations of several mild stressors, or by mutations in genes such as *AtDDM1* that are involved in the epigenetic control of transposable elements (Quadrana and Colot, 2016).

## Supplementary data

Supplementary data are available at *JXB* online.

Fig. S1. Genome-wide DNA methylation patterns between leaves of stressed and non-stressed plants.

Fig. S2. Chromosomal distribution of local gains and losses of DNA methylation between leaves of stressed and non-stressed plants.

Fig. S3. Correlation of differential methylation with changes in gene expression in response to mild drought.

Fig. S4. Increased methylome instability in the progenies of stressed lines.

Table S1. Estimates of trait-based maternal effects in P3 for each accession.

Table S2. Estimates of trait-based maternal effects in P4 for each accession.

Table S3. Estimates of individual within-line variances in P3 for models with trait-based maternal effects for each accession.

Table S4. Estimates of individual within-line variances in P4 for models with trait-based maternal effects for each accession.

Table S5. Summary statistics of whole-genome bisulfite sequencing data.

Table S6. Total fraction of methycytosines and their distribution in each of the three types of site (CG, CHG, CHH).

Table S7. List of differentially methylated positions (DMPs).

Table S8. List of differentially methylated regions (DMRs).

Table S9. List of differentially expressed genes.

Table S10. Gene ontology analysis for plastic gene expression responses.

eraa132_suppl_supplementary_Figures_S1_S4_Tables_S1_S6

eraa132_suppl_supplementary_Table_S7

eraa132_suppl_supplementary_Table_S8

eraa132_suppl_supplementary_Table_S9

eraa132_suppl_supplementary_Table_S10
