# Supplementary material for: Mild drought in the vegetative stage induces phenotypic, gene expression, and DNA methylation plasticity in Arabidopsis but no transgenerational effects
Source: J Exp Bot. 2020 Mar 13;71(12):3588–602. doi: 10.1093/jxb/eraa132 (PMC7307858; doi:10.1093/jxb/eraa132)
Supplement: eraa132_suppl_supplementary_Figures_S1_S4_Tables_S1_S6 [file eraa132_suppl_supplementary_figures_s1_s4_tables_s1_s6.pdf]

Supplementary material with

Mild drought in the vegetative stage induces phenotypic, gene expression and DNA methylation plasticity in Arabidopsis but no transgenerational effects

Running title: Assessing transgenerational effects in Arabidopsis

Tom JM Van Dooren, Amanda Bortolini Silveira, Elodie Gilbault, José M. Jiménez-Gómez, Antoine Martin, Liên Bach, Sébastien Tisné, Leandro Quadrana, Olivier Loudet and Vincent Colot

Figures S1-S4, Tables S1-S6.

Fig. S1. Genome-wide DNA methylation patterns are similar between leaves of stressed and non-stressed plants. (a) Global percentage of methylated (mC) and non-methylated (nmC) cytosines over covered positions (≥3 reads). (b) Fraction of mCs in each sequence context. (c) Distribution of CG, CHG and CHH DNA methylation patterns across genes, transposable elements and intergenic regions.

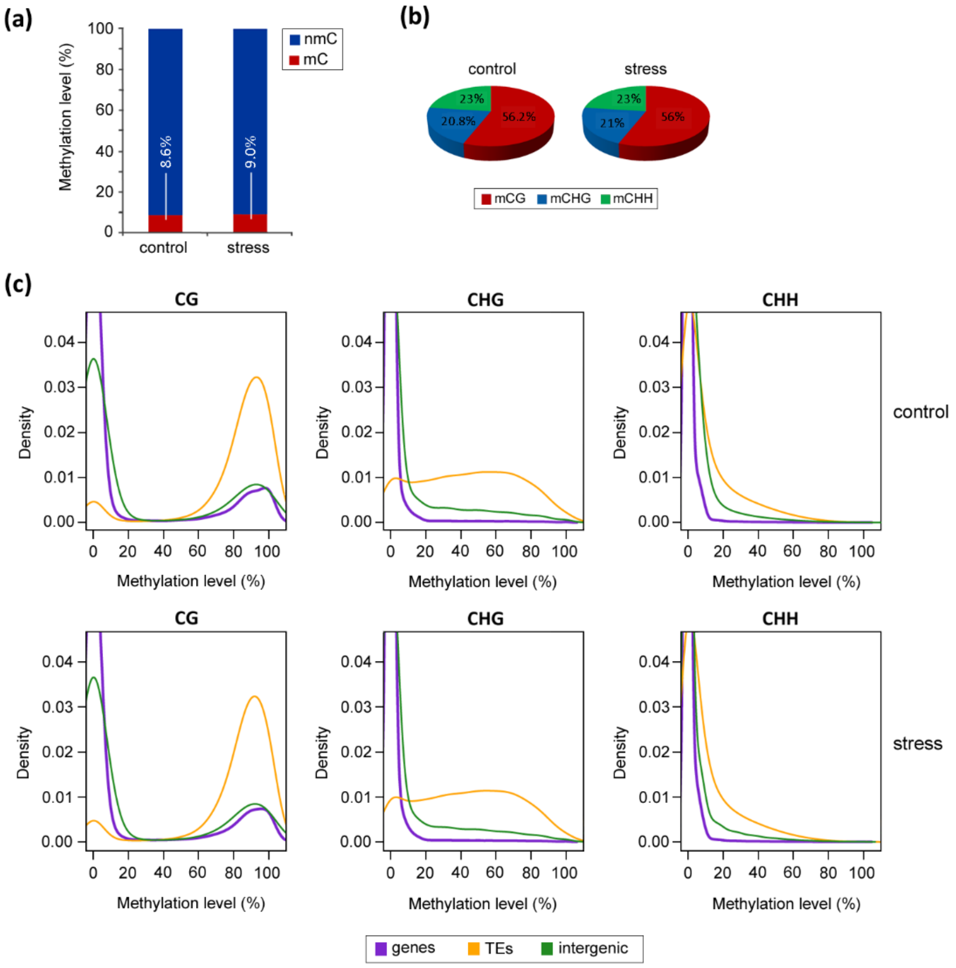

Fig. S2. Chromosomal distribution of local gains and losses of DNA methylation found between leaves of stressed and non-stressed plants. Dashed vertical lines delimit centromeric and pericentromeric regions.

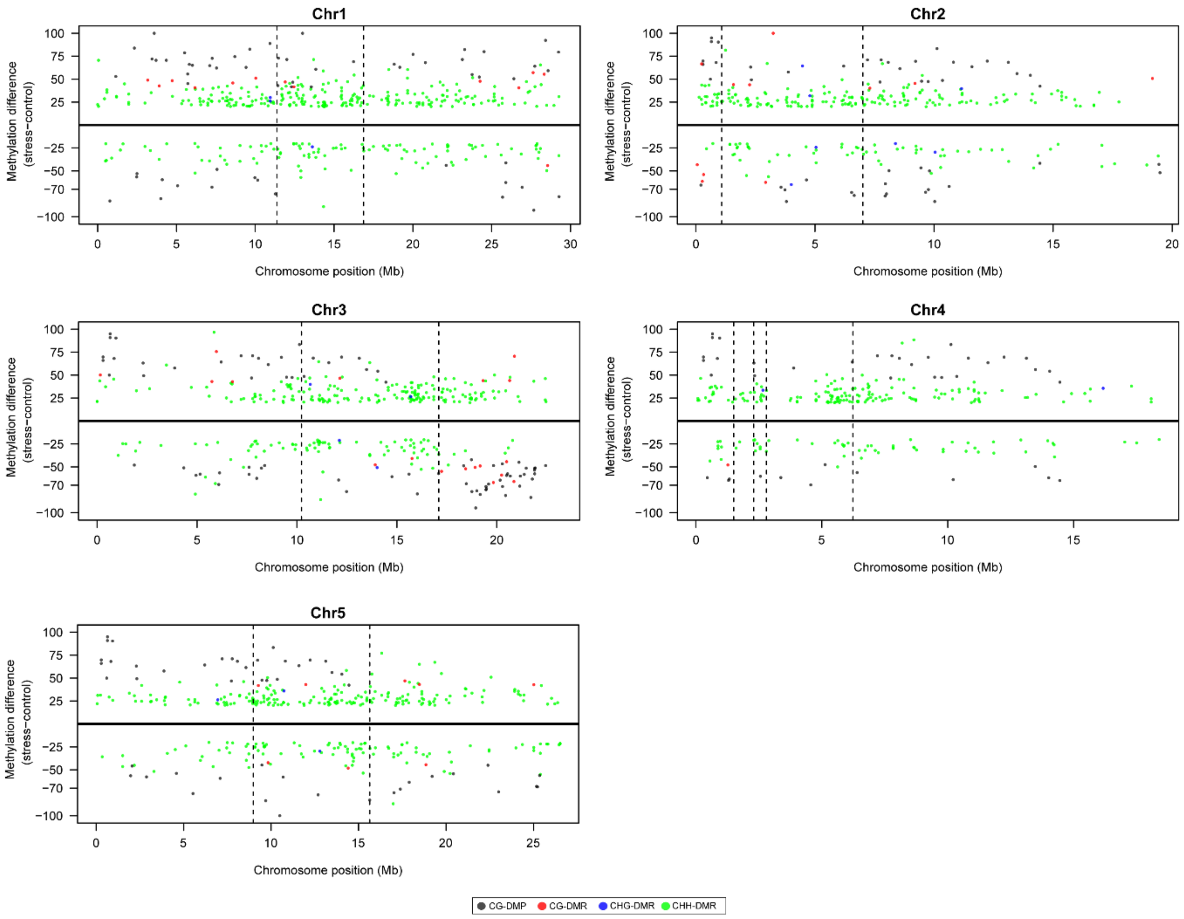

Fig. S3. Differential methylation is hardly congruent to changes in gene expression in response to mild drought. (a) Distribution of distances relative to the closest DMR for the 468 genes responsive to mild drought and a random set of genes. (b) Genes located less than 500bp from DMR that are differentially expressed in response to mild drought.

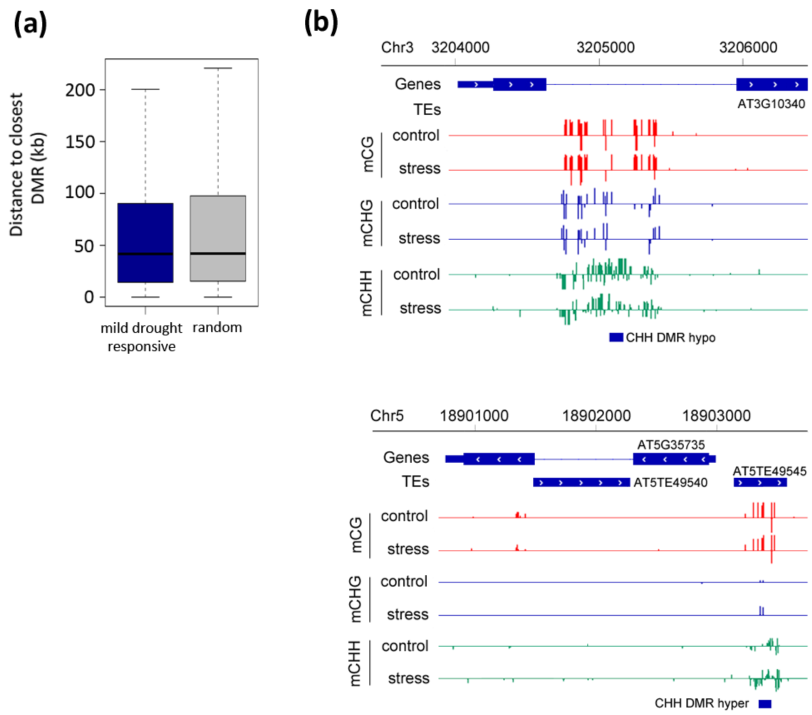

Fig. S4. The progenies of stressed lines exhibit increased methylome instability.
Distribution of variance differences in local DNA methylation patterns (100bp windows) between the five G3 lines with control or stress history.

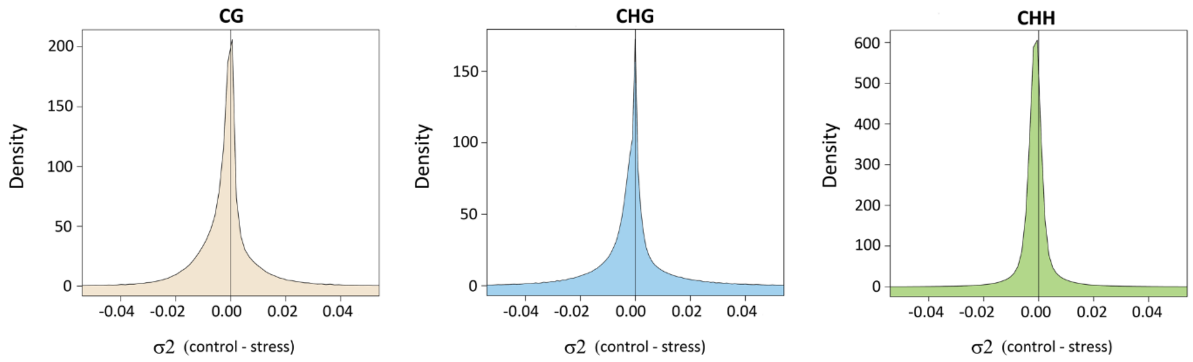

Table S1. Estimates of trait-based maternal effects in P3 per accession. For each trait in offspring, significant maternal trait effects are listed in the same order each time per accession and offspring trait. Estimates and standard errors are given, plus an indication of the tail probability of the LRT test for the effect (*: p < 0.05, **: p < 0.01, ***: p < 0.001). Effects of maternal traits on the same trait in offspring are shown in bold, which are candidates for direct and persistent maternal effects. When the slope of the maternal trait varies between the control, Memory (M) and Drought (D) groups, estimates per groups are given. We had insufficient parental individuals to fit M:D interactions on trait slopes well.

	Offspring	Log(PRA)	Log(CircleRadius)	Log(Compactness)	RedMean	BlueMean
Col-0	Log(PRA)<br>CircleRadius<br>Compactness<br>RedMean<br>BlueMean	-<br>-<br>-<br>-	-<br>-<br>-<br>-<br>-	-<br>-<br>-<br>-<br>-	-<br>-<br>-<br>-<br>-	-<br>-<br>-<br>-<br>-
Bur	Log(PRA)<br>CircleRadius<br>Compactness<br>RedMean<br>BlueMean	-<br>-<br>C: [-3.31,-0.36] M: [1.10,3.00] ***<br>-<br>-	-<br>-<br>C: [0.36,1.84] M: [-0.95,0.01]***<br>-<br>-	C: [-1.02,-0.01] M:[-3.11,-1.07]**<br>C:[-0.18,1.43] M:[2.21,6.22]**<br>**C:[-0.06,0.80] M:[3.13,8.32]*****<br>C:[-4.13,0.30] M:[1.78,12.86]*<br>[2.14,12.55]**	-<br>-<br>-<br>-<br>-	C: [0.01,0.05] D: [-0.08,0.01]**<br>C: [-0.11,-0.02] D:[-0.01,0.16]**<br>[-0.09,-0.17]**<br>-<br>-
Cvi	Log(PRA)<br>CircleRadius<br>Compactness<br>RedMean<br>BlueMean	-<br>-<br>-<br>-<br>-	-<br>-<br>-<br>-<br>-	-<br>-<br>-<br>-<br>-	-<br>-<br>-<br>-<br>-	-<br>-<br>-<br>-<br>-
Sha	Log(PRA)<br>CircleRadius<br>Compactness<br>RedMean<br>BlueMean	-<br>-<br>-<br>-<br>-	-<br>-<br>-<br>-<br>-	-<br>[0.05,0.22]**<br>-<br>-<br>-	-<br>-<br>-<br>-<br>-	-<br>-<br>-<br>-<br>-
Tsu	Log(PRA)<br>CircleRadius<br>Compactness<br>RedMean<br>BlueMean	-<br>-<br>-<br>-<br>-	-<br>-<br>-<br>-<br>-	-<br>-<br>-<br>-<br>-	-<br>-<br>-<br>-<br>-	-<br>-<br>C: [-0.27,-0.03] M[-0.01,0.21]**<br>-<br>-

Table S2. Estimates of trait-based maternal effects in P4 per accession. For each trait in offspring, significant maternal trait effects are listed in the same order each time per accession and offspring trait. Estimates and standard errors are given, plus an indication of the tail probability of the LRT test for the effect (*: p < 0.05, **: p < 0.01, ***: p < 0.001). Effects of maternal traits on the same trait in offspring are shown in bold, which are candidates for direct and persistent maternal effects. When the slope of the maternal trait varies between the control, Memory (M) and Drought (D) groups, estimates per groups are given. We had insufficient parental individuals to fit M:D interactions on trait slopes well.

	Offspring	Log(PRA)	Log(CircleRadius)	Log(Compactness)	RedMean	BlueMean
Col-0	Log(PRA)	-	-	-	-	-
	CircleRadius	-	-	-	-	-
	Compactness	-	-	**C:[-0.36,0.02] M:[0.31,2.0]****	-	-
	RedMean	-	-	-	-	-
	BlueMean	-	-	-	-	-
Bur	Log(PRA)	-	-	-	-	-
	CircleRadius	-	-	-	-	
	Compactness	-	-	**[0.03,0.67]***	-	-
	RedMean	-	-	-	-	-
	BlueMean	-	-	-	-	-
Cvi	Log(PRA)	-	-	-	-	-
	CircleRadius	-	-	-	-	-
	Compactness	-	C:[-2.1,2.1] D:[0.4,4.6]* M:[-6.4,-1.1]**	-	-	C:[0.02,0.10] D:[-0.05,0.02]**
	RedMean	-	-	-	-	-
	BlueMean	-	-	-	-	-
Sha	Log(PRA)	-	-	-	-	-
	CircleRadius	-	-	-	-	-
	Compactness	-	-	-	-	-
	RedMean	-	-	-	-	-
	BlueMean	[-264, -1] *	-	-	-	-
Tsu	Log(PRA)	-	-	-	-	-
	CircleRadius	-	-	-	-	-
	Compactness	-	-	-	-	-
	RedMean	-	-	-	-	-
	BlueMean	-	-	-		-

Table S3. Estimates of Individual within-line variances in P3 for models with trait-based maternal effects per accession. Estimates of standard deviations are given, plus an indication of the tail probability of the LRT test for the effect (*: $p < 0.05$, **: $p < 0.01$, ***: $p < 0.001$). In many cases standard errors of the estimates could not be obtained. When the variances in the control, Memory (M, ancestral drought) and Drought (D) groups differ, estimates of standard deviations are given per group. The three cases where the variance in the memory group is smaller than in the control are shown in bold. Residual standard deviationss in the Control treatment are given for comparison.

	PRA		CircleRadius		Compactness		RedMean		BlueMean	
Acces sion	Line	Residual (C)	Line	Residual (C)	Line	Residual (C)	Line	Residual (C)	Line	Residual (C)
Col-0	C: 0.18<br>D: 0.29***	0.02	C: 0.16<br>D: 0.10***	0.03	**C: 0.04**<br><br>**M: 0.02*** **	0.02	**C: 0.04**<br><br>**M: 0.01*** **	0.01	**C: 0.013**<br><br>**M: 0.004*** **	0.003
Bur	0.13	0.03	0.07	0.02	0.03	0.02	C: 0.01<br>D: 0.04***	0.01	C: 0.004<br>M: 0.008***	0.002
Cvi	C: 0.14<br>M: 0.62***	0.02	C: 0.08<br>M: 0.26***	0.02	0.04	0.03	C: 0.01<br>M: 0.07***	0.01	C: 0.002<br>M: 0.011***	0.002
Sha	0.17	0.02	0.09	0.02	0.02	0.02	C: 0.01<br>D: 0.09***	0.01	0.010	0.001
Tsu	C: 0.10<br>D: 0.22***	0.03	C: 0.04<br>D: 0.12***	0.02	0.02	0.02	C: 0.05<br>**M: 0.01*** **	0.01	0.007	0.002

Table S4. Estimates of Individual within-line variances in P4 for models with trait-based maternal effects per accession. Estimates are given, plus an indication of the tail probability of the LRT test for the effect (*: p < 0.05, **: p < 0.01, ***: p < 0.001). In many cases standard errors could not be obtained. When the variances in the control, Memory (M) and Drought (D) groups differ, estimates of standard deviations per group are given. The two cases where the variance in the memory group is smaller than in the control are shown in bold. Residual variances are given for comparison.

	PRA		CircleRadius		Compactness		RedMean		BlueMean	
Accession	Line	Residual (C)	Line	Residual (C)	Line	Residual (C)	Line	Residual (C)	Line	Residual (C)
Col-0	0.15	0.05	0.078	0.02	0.04	0.03	0.008	0.006	0.006	0.004
Bur	C 0.31<br>D 1.22***	0.03	C: 0.16<br>D: 0.53***	0.03	C: 0.03<br>D: 0.10***	0.02	C: 0.005<br>D: 0.011***	0.005	C: 0.002<br>D: 0.016***	0.002
Cvi	C 0.18<br>M 0.75***	0.02	C: 0.12<br>M: 0.35***	0.03	C: 0.05<br>D: 0.10**	0.04	C: 0.013<br>M: 0.020*	0.008	C: 0.004<br>M: 0.008***	0.002
Sha	1.56	0.07	C: 0.36<br>D: 1.08 ***	0.06	**C: 0.36**<br>**M: 0.09*****	0.11	C: 0.011<br>D: 0.024***	0.005	C: 0.008<br>D: 0.035***	0.002
Tsu	C: 0.27<br>M: 1.22***	0.03	C: 0.14<br>D: 0.67 ***	0.06	C: 0.02<br>D: 0.14***		C: 0.010<br>D: 0.017 **	0.006	C: 0.006<br>D: 0.016***	0.003

Table S5. Summary statistics of whole genome bisulfite sequencing data

Sample name	Total nº of read pairs	% of pairs uniquely mapping to the genome	% duplication	Nº of non-redundant pairs uniquely mapping to the genome	Bisulfite non-conversion rate CG-CHG-CHH	% of cytosines covered by ≥3 ≤100 reads	Average cytosine coverage per strand (+/-) CG-CHG-CHH
Control	27222162	66.5%	4.2%	17305907	0.41-0.42-0.45	86.1%	15.1/15.2 - 13.9/14.1 - 10.9/11
Stress	25053147	65.9%	4.2%	15707311	0.35-0.37-0.40	79.6%	13.6/13.7 - 12.5/12.7 - 9.7/9.8
C_1C_2-control_4	43852152	68.4%	4.8%	28568320	0.29-0.31-0.34	89.1%	25.6/26 - 24.3/24.8 - 18.5/18.7
C_1C_2-control_128	47709742	66.5%	5.2%	30076257	0.69-0.71-0.77	93.4%	26.5/27 - 25.3/25.8 - 20.1/20.3
C_1C_2- control_366	46683764	65.8%	6.8%	28647436	0.90-0.92-0.99	94.3%	24.5/25 - 23.4/23.8 - 19.3/19.6
C_1C_2- control_482	47698418	62.5%	5.2%	28236081	0.56-0.58-0.62	93.1%	24.6/25 - 23.5/24 - 18.8/19.1
C_1C_2- control_490	41903141	66.1%	4.9%	26362560	0.34-0.35-0.37	93.9%	22.2/ 22.5 - 21.8/22.1 - 17.9/18.1
S_1S_2 – control_17	47940820	67.8%	4.9%	30918979	0.38-0.39-0.43	91.7%	27.5/27.9 - 26.2/26.7 - 20.2/20.5
S_1S_2 – control_19	46390271	69.5%	5.4%	30517756	0.33-0.35-0.37	94.2%	26/26.3 - 25.1/25.6 - 20.5/20.8
S_1S_2 – control_23	41766897	68.3%	4.2%	27337030	0.36-0.48-0.41	91.5%	24.0/24.7 - 23/23.4 - 18/18.3
S_1S_2 – control_375	43558852	63.0%	5.2%	26019818	0.46-0.48-0.52	90.4%	23.2/23.5 - 25.4/22.4 - 17.4/17.6
S_1S_2 – control_501	49209817	64.0%	5.5%	29755708	0.27-027-0.30	91.8%	26.9/27.2 - 24.4/25.8 - 19.4/19.7

Table S6. Total fraction of methycytosines and distribution in each sequence context

Sample name	% of mC	%mCG	%mCHG	%mCHH
Control	8.6	56.2	20.9	23
Stress	9.1	55.9	21	23.1
C_1C_2-control_4	8.2	55.7	22.2	22.1
C_1C_2-control_128	8.2	54.6	21.5	23.9
C_1C_2- control_366	8.3	53	21.5	25.5
C_1C_2- control_482	8.4	54	21.8	24.2
C_1C_2- control_490	6.5	56.7	20.9	22.4
S_1S_2-control_17	8	56.2	21.9	21.9
S_1S_2- control_19	7.1	56.7	21.4	21.9
S_1S_2-control_23	7.9	56.2	22	21.8
S_1S_2-control_375	8	55.1	21.8	23.1
S_1S_2-control_501	8.5	56	22.3	21.7
